# Supplementary figures and images for: Specific Upregulation of TRPC1 and TRPC5 Channels by Mineralocorticoid Pathway in Adult Rat Ventricular Cardiomyocytes
Source: Cells. 2019 Dec 23;9(1):47. doi: 10.3390/cells9010047 (PMC7017140; doi:10.3390/cells9010047)

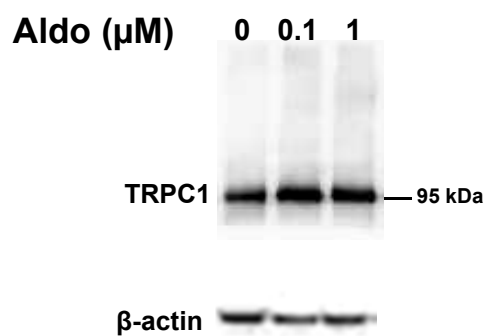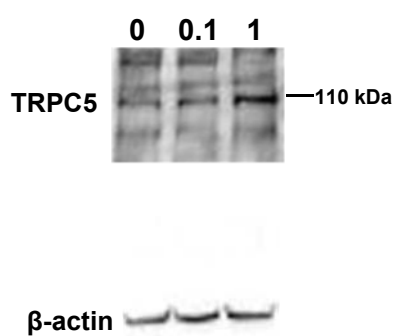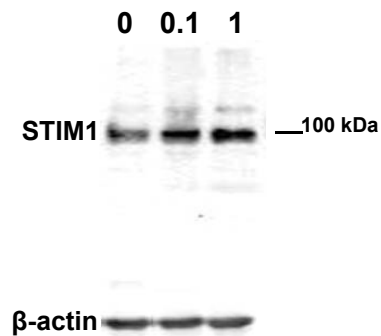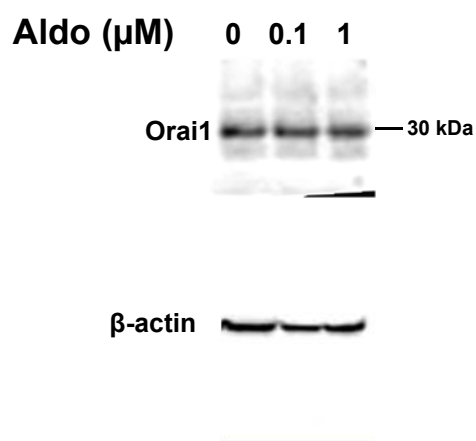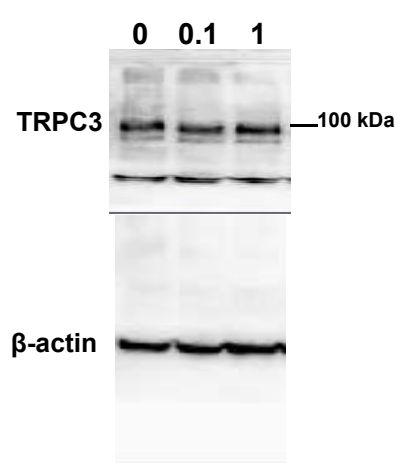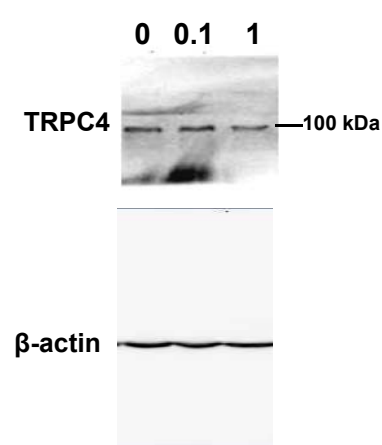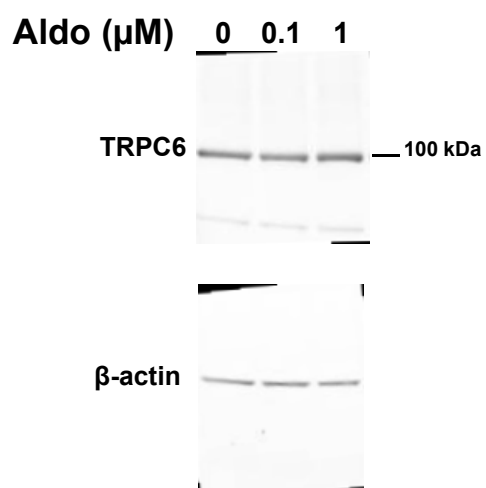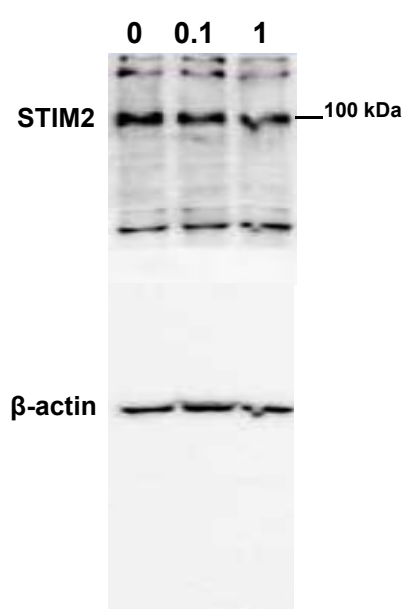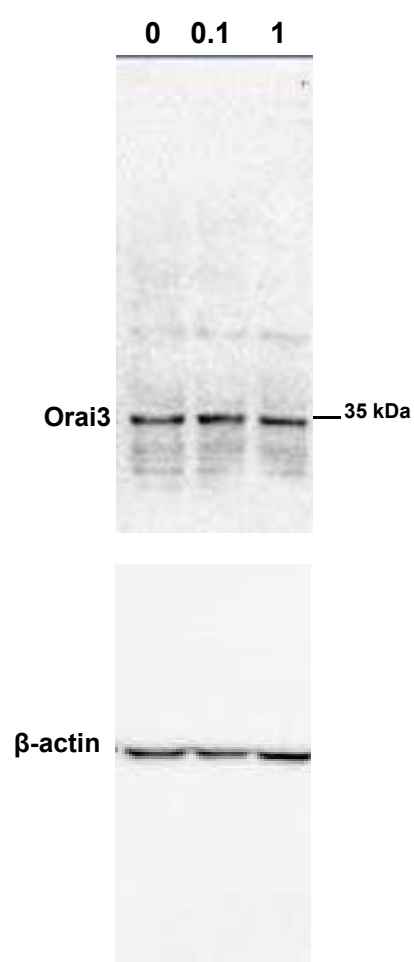

Supplemental Figure 1

Supplement: Supplementary file 1 [file cells-09-00047-s001.pdf]
